# A novel inflammation-related prognostic model for predicting the overall survival of primary central nervous system lymphoma: A real-world data analysis

**DOI:** 10.3389/fonc.2023.1104425

**Published:** 2023-03-28

**Authors:** Zhentian Wu, Chenyi Wang, Yao Lyu, Zheshen Lin, Ming Lu, Shixiong Wang, Bingxuan Wang, Na Yang, Yeye Li, Jianhong Wang, Xiaohui Duan, Na Zhang, Jing Gao, Yuan Zhang, Miaowang Hao, Zhe Wang, Guangxun Gao, Rong Liang

**Affiliations:** ^1^Department of Hematology, Xijing Hospital, Air Force Medical University, Xi’an, Shaanxi, China; ^2^Department of Geriatrics, Xijing Hospital, Air Force Medical University, Xi’an, Shaanxi, China; ^3^Department of Hematology, Tangdu Hospital, Air Force Medical University, Xi’an, Shaanxi, China; ^4^Department of Respiratory, Tangdu Hospital, Air Force Medical University, Xi’an, Shaanxi, China; ^5^Department of Pathology, Xijing Hospital, Air Force Medical University, Xi’an, Shaanxi, China

**Keywords:** PCNSL, prognostic model, nomogram, risk stratification, SIRI

## Abstract

**Background:**

Primary central nervous system lymphoma (PCNSL) is a type of extranodal non-Hodgkin lymphoma. Although there are widely used prognostic scores, their accuracy and practicality are insufficient. Thus, a novel prognostic prediction model was developed for risk stratification of PCNSL patients in our research.

**Methods:**

We retrospectively collected 122 patients with PCNSL from two medical centers in China from January 2010 to June 2022. Among them, 72 patients were used as the development cohort to construct a new model, and 50 patients were used for the validation. Then, by using univariate and multivariate Cox regression analsis and Lasso analysis, the Xijing model was developed and composed of four variables, including lesion number, β2-microglobulin (β2-MG), systemic inflammation response index (SIRI) and Karnofsky performance status (KPS). Finally, we evaluated the Xijing model through internal and external validation.

**Results:**

Compared with the original prognostic scores, the Xijing model has an overall improvement in predicting the prognosis of PCNSL according to the time-dependent area under the curve (AUC), Harrell’s concordance index (C-index), decision curve analysis (DCA), integrated discrimination improvement (IDI) and continuous net reclassification index (NRI). For overall survival (OS) and progression-free survival (PFS), the Xijing model can divide PCNSL patients into three groups, and shows more accurate stratification ability. In addition, the Xijing model can still stratify and predict prognosis similarly better in the elderly with PCNSL and subgroups received high-dose methotrexate (HD-MTX) or Bruton’s tyrosine kinase inhibitors (BTKi). Finally, external validation confirmed the above results.

**Conclusions:**

Integrating four prognostic factors, including imaging findings, tumor burden, systemic inflammation response index, and comprehensive physical condition, we provided a novel prognostic model for PCNSL based on real-world data and evaluated its predictive capacity.

## 1 Introduction

Primary central nervous system lymphoma (PCNSL) is a rare extranodal non-Hodgkin lymphoma, which accounts for approximately 3-4% of intracranial malignancies and is localized to the cerebral parenchyma, leptomeninges, spinal cord and eyes, without peripheral involvement (1, 2). About 95% of PCNSL pathological types are diffuse large B-cell lymphoma (DLBCL), and the remaining rare pathological types include T-cell, Burkitt, lymphoblastic, and marginal zone lymphomas (3–6). PCNSL is characterized by strong aggressiveness, rapid disease progression, and poor prognosis, and the overall survival time of untreated PCNSL patients is only 1.5 months (7). Therefore, risk stratification and prognostic prediction of PCNSL patients are particularly important.

At present, the most widely used prediction models are the IELSG prognostic score and the MSKCC prognostic score. The former model, developed by the International Extranodal Lymphoma Study Group, contains five variables, age, deep brain lesions, Eastern Cooperative Oncology Group (ECOG) performance status, lactate dehydrogenase (LDH), and cerebrospinal fluid (CSF) protein level, each assigned 1 score, thus classifying PCNSL patients into low-risk (0-1 score), medium-risk (2-3 score), and high-risk (4-5 score) groups (8).. The latter was proposed by Memorial Sloan-Kettering Cancer Center and consists of only two variables: age and Karnofsky performance status (KPS). Patients can be divided into three groups according to age ≤ 50 years old, age>50 years old and KPS≥70, and age>50 years old and KPS<70 (9).

In recent years, the treatment of PCNSL is mainly based on high-dose methotrexate (HD-MTX). With the development of new therapeutic strategies such as immunotherapy for novel molecular targets, autologous hematopoietic stem cell transplantation, and CART therapy, the PFS and OS of patients with PCNSL have been improved (10–14), which may lead to changes in the predictive efficacy of previously developed IELSG and MSKCC scores. Therefore, there is an urgent need for a reliable predictive model suitable for the current stage to predict the survival outcome of PCNSL patients, carry out fine risk stratification, and provide a basis for clinical decision-making.

In this retrospective study, we collected data from 122 patients with PCNSL from two medical centers in Northwest China to construct a new predictive model, which was externally validated. The newly constructed model can more accurately predict the prognosis of PCNSL patients, stratify the risk of patients and provide clinical decision-making guidance.

## 2 Materials and methods

### 2.1 Cohort selection

**Figure 1:**
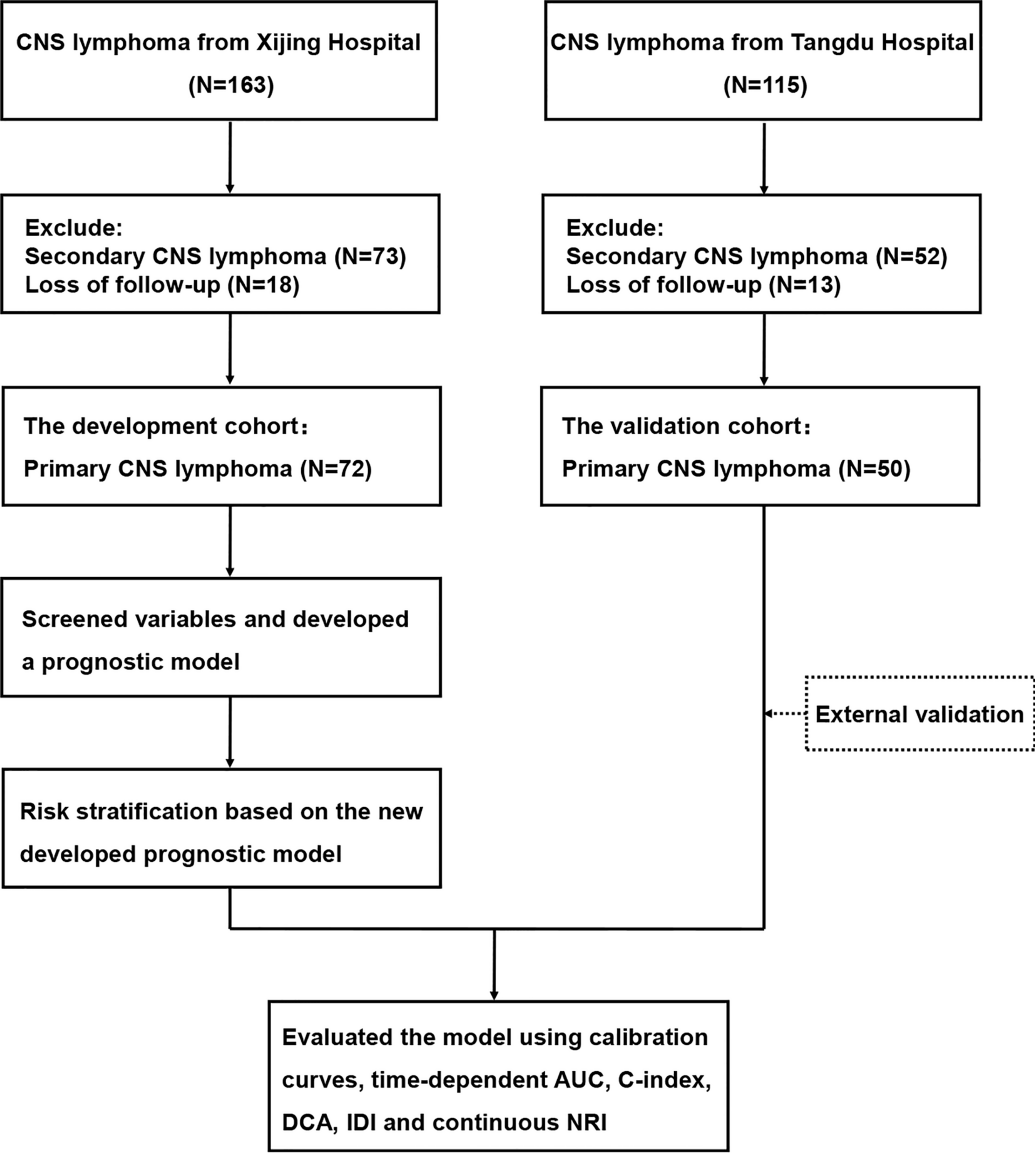
Flow chart of patient enrollment.

A total of 122 newly diagnosed PCNSL patients were retrospectively collected from Xijing Hospital and Tangdu Hospital from January 2010 to June 2022. The diagnostic criteria were according to the 2003 Classification of Tumors of the Central Nervous System 2021, and no peripheral involvement was found by PET-CT, bone marrow cell morphology and puncture biopsy (15). The follow-up time was up to June 2022. All patients were at least 18 years old, and treatment and survival data were available. We selected PCNSL patients in Xijing Hospital as the development cohort (N=72), and PCNSL patients in Tangdu Hospital as the validation cohort (N=50). The flow chart of patient inclusion is shown in **Figure 1**.

### 2.2 Data collection

Basic and specific clinical information of patients was collected when PCNSL was first diagnosed. Patients’ basic characteristics include age, sex, and history of underlying diseases, while specific clinical characteristics contain peripheral blood neutrophil count (NEU, ×10^9^/L), lymphocyte count (LYM, ×10^9^/L), mononuclear cell count, (MONO, ×10^9^/L), platelet count (PLT, ×10^9^/L), β_2_-microglobulin (β_2_-MG, mg/L), albumin (ALB, g/L), LDH (IU/L), CSF protein (g/L), KPS, ECOG performance status (ECOG-PS), number and location of lesions, immunohistochemical (IHC) results of pathologic tissue, IELSG and MSKCC score. In addition, immune inflammation index and prognostic nutritional index were calculated respectively based on β2-MG, LDH, ALP, ALB, and complete blood cell count for new prognostic model development, including systemic inflammation response index (SIRI), systemic immune inflammation index (SII), neutrophil-to-lymphocyte ratio (NLR), platelet-to-lymphocyte ratio (PLR), β_2_-microglobulin-to-lymphocyte ratio (βLR), lactate dehydrogenase-to-lymphocyte ratio (LLR), lymphocyte-to-monocyte ratio (LMR), serum albumin-to-alkaline phosphatase ratio (AAPR) and prognostic nutritional index (PNI). Their calculation formula is as follows (7): SIRI= NEU count ×MONO count/LYM count, SII= PLT count ×NEU count/LYM count, NLR= NEU count/LYM count, PLR= PLT count/LYM count, βLR= β2-MG/LYM count, LLR= LDH/LYM count, LMR= LYM count/MONO count, AAPR= ALB/ALP, PNI= ALB+5×LYM count.

### 2.3 Ending event definitions

The last follow-up was up to June 2022. The primary end point event was overall survival (OS), defined as the time from the diagnosis of PCNSL to all-cause death or the last follow-up. The secondary end point was progression-free survival (PFS), defined as the time from the diagnosis of PCNSL to disease progression or all-cause death or until the last follow-up.

### 2.4 Variables selection

Before screening the variables used to construct the new model, continuous variables with reference ranges are transformed into categorical variables according to normal values, while other continuous variables without normal reference ranges are transformed to categorical variables based on calculated cut-off values. As for the cut-off values of pathological indicators, we refer to previous published papers (16, 17). Besides, we perform multivariate imputation on missing data.

Univariate Cox regression was utilized to analyze and evaluate the variables in the development cohort, and P<0.1 was used as the criterion for screening candidate variables. To prevent overfitting, we performed Lasso regression on the selected candidate variables (18). Considering the clinical practicality, we finally selected four variables for the construction of a new predictive model for PCNSL, and wholly evaluated the model by multivariate Cox regression analysis.

### 2.5 Validation of the new developed model

We conducted internal and external validation of the developed model respectively, and adopted the following indicators to evaluate and verify the predictive ability of the model in the development and validation cohort. (1) Time-dependent area under the curve (AUC) and Harrell’s concordance index (C-index): The discrimination of the new model was tested by time-dependent AUC of the receiver operator characteristic (ROC) and C-index (19). (2) Calibration curve: Bootstrap was used to conduct 1000 times resamples to draw the calibration curve. The coincidence degree between the curve and the 45° diagonal reflects the degree of agreement between the predicted probability and the actual result (20). (3) Decision curve analysis (DCA): It reflects the clinical usefulness of the new model as well as the range of risk thresholds and net benefits, which shows if the model was the best choice for patients with PCNSL (21). (4) Integrated discrimination improvement (IDI) and continuous net reclassification index (NRI): These two indicators reflect whether the predictive capacity of the new model is improved compared with the original IELSG and MSKCC scores (22, 23).

### 2.6 Statistical methods

R version 4.1.0 and SPSS version 26.0 were used for statistical analysis, and a two-sided P<0.05 is statistically significant. Qualitative variables were analyzed by chi-square test or Fisher exact test, and quantitative variables were analyzed by Mann-Whitney U test. Kaplan-Meier method was used to draw survival curves, and Log-rank was used to test the differences between groups. Besides, univariate and multivariable Cox proportional hazard models were used to assess the prognostic variables and calculate hazard ratios (HR) with 95% confidence intervals (CI). The R packages used in the above statistical analysis are detailed in the Supplementary Materials.

## 3 Results

### 3.1 Baseline characteristics

The median ages of patients in the development cohort (N=72) and validation cohort (N=50) are 57.5(48.25-63) years and 61(51-67) years, respectively, and the male-to-female ratios are 1.32:1 and 1.08:1, respectively. In all collected patients, most of patients received chemotherapy, and other patients received treatments including surgery, whole brain radiation therapy (WBRT), surgery combined with WBRT, and palliative care. Among them, 46 patients (63.9%) in the development cohort received HD-MTX-based chemotherapy regimen (MTX or R ± MA) and 23 patients (31.9%) received immuno-targeted therapy containing Bruton’s tyrosine kinase inhibitors (BTKi). Similarly, the number and proportion of patients in the validation cohort are 28(56%) and 10(20%), respectively. The remaining baseline characteristics are shown in **Table 1**. The duration of follow-up is 138 months as of June 1, 2022, with a median follow-up of 40 months (range from 1 to 126 months) and 48 months (range from 3 to 90 months) for the development and validation cohort. The median OS is 21 months and 17 months, and the median PFS is 6 months and 7 months, respectively.

**Table 1:** Baseline characteristics of development and validation cohorts.

Characteristics	Development (n=72) n (%)	Validation (n=50) n (%)	P
Patient specific			
Age>60	46 (63.9)	30 (60)	0.663
Median age (IQR)	57.5 (48.25-63)	61 (51-67)	0.067
Male	41 (56.9)	26 (52)	0.589
Underlying disease			
Hypertension	22 (30.6)	13 (26)	0.584
Diabetes	13 (18.1)	8 (16)	0.767
CHD	5 (6.9)	2 (4)	0.492
Disease specific			
Deep brain lesions	43 (59.7)	37 (26)	0.103
Multiple lesions	43 (59.7)	28 (56)	0.682
ECOG-PS≥2	62 (86.1)	39 (78)	0.243
Median KPS (IQR)	60 (50-70)	60 (50-70)	0.685
CSF protein			0.740
>0.45 g/L	37 (51.4)	30 (60)	
Missing	7 (9.7)	—	
LDH>250 IU/L	19 (26.4)	22 (44)	0.043
β_2_-MG>2.5 mg/L	42 (58.3)	32 (64)	0.529
D-Dimer>0.6 mg/L	43 (59.7)	32 (64)	0.633
RDW>0.15	16 (22.2)	19 (38)	0.058
ALB≥40 g/L	56 (77.8)	25 (50)	0.001
Bcl-2≥60%	37 (51.4)	24 (48)	0.713
Bcl-6≥40%	32 (44.4)	26 (52)	0.411
C-myc≥40%	54 (75)	35 (70)	0.541
MUM1≥40%	22 (30.6)	20 (40)	0.280
Ki67≥80%	50 (69.4)	37 (74)	0.584
SIRI≥3.3	19 (26.4)	14 (28)	0.844
SII≥97.4	17 (23.6)	33 (66)	<0.001
NLR≥5.1	29 (40.3)	18 (36)	0.633
PLR≥78.9	62 (86.1)	46 (92)	0.316
LLR≥220.3	24 (33.3)	17 (34)	0.939
βLR≥4.1	10 (13.9)	8 (16)	0.746
LMR≥2.7	48 (66.7)	29 (58)	0.329
PNI≥40	45 (62.5)	33 (66)	0.393
AAPR≥0.6	31 (43.1)	13 (26)	0.054
IELSG stratification			0.015
Low-risk	13 (18.1)	12 (24)	
Median-risk	35 (48.6)	14 (28)	
High-risk	17 (23.6)	24 (48)	
Missing	7 (9.7)	—	
MSKCC stratification			0.507
Low-risk	21 (29.2)	10 (20)	
Median-risk	19 (26.4)	14 (28)	
High-risk	32 (44.4)	26 (52)	
Chemotherapy regimen			
HD-MTX-based	46 (63.9)	28 (56)	0.380
Treatment containing BTKi	23 (31.9)	10 (20)	0.144
AHSCT	5 (6.9)	—	—

IQR, interquartile range; CHD, coronary heart disease; ECOG-PS, Eastern Cooperative Oncology Group performance status; KPS, Karnofsky performance status; CSF, cerebrospinal fluid; LDH, lactate dehydrogenase; β2-MG, β2-microglobulin; RDW, red blood cell volume distribution width; ALB, albumin; SIRI, systemic inflammation response index; SII, systemic immune inflammation index; NLR, neutrophil-to-lymphocyte ratio; PLR, platelet-to-lymphocyte ratio; LLR, lactate dehydrogenase-to-lymphocyte ratio; βLR, β2-microglobulin-to-lymphocyte ratio; LMR, lymphocyte-to-monocyte ratio; PNI, prognostic nutritional index; AAPR, serum albumin-to-alkaline phosphatase ratio; IELSG, International Extranodal Lymphoma Study Group; MSKCC, Memorial Sloan Kettering Cancer Center; HD-MTX, high-dose methotrexate; BTKi, Bruton’s tyrosine kinase inhibitors; AHSCT, Autologous haematopoietic stem cell transplantation; -, none.

### 3.2 Development and evaluation of the Xijing model

**Figure 2:**
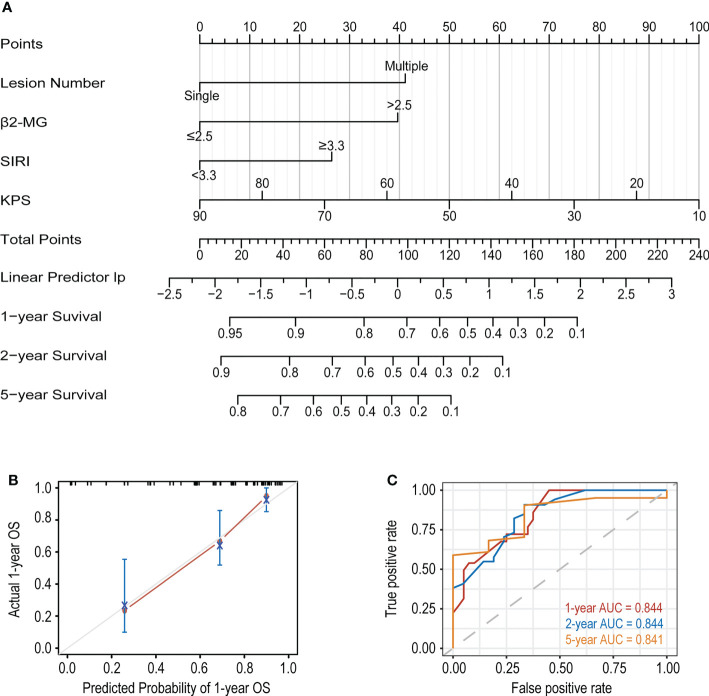
**(A)** The nomogram based on the development cohort to predict OS of PCNSL patients. **(B)** The calibration curve of the Xijing model for predicting 1-year OS. **(C)** The 1, 2 and 5-year ROC curves of the Xijing model.

We first performed univariate Cox regression analysis on all the variables in the development cohort and filtered out 16 variables with P<0.1, including age, number of lesions, ECOG-PS≥2, KPS, LDH, β2-MG, ALB, RDW, D-Dimer, Bcl-2, C-myc, SIRI, PLR, LLR, βLR, and PNI (**Table 2**). Lasso analysis was then performed on above variables to identify six candidate variables: age, Bcl-2, number of lesions, β2-MG, KPS, and SIRI (**Supplemental Figure S1**). However, based on clinical experience, two candidate variables, age and Bcl-2, were excluded, and we then determined four variables, number of lesions, β2-MG, KPS and SIRI, to be included in the multivariate Cox regression analysis. Finally, a new prediction model was constructed by multivariate Cox proportional hazard model (**Table 3**), in which the P values of the three tests (Likelihood ratio, Wald, and Score) of the model were all less than 0.001, indicating a good fit of the Xijing model. The nomogram of the Xijing model is shown in **Figure 2A**, of which the points of each variable and the 1-year, 2-year and 5-year survival probability corresponding to the total points in the nomogram are displayed in **Supplemental Tables S1**; **S2**.

**Table 2:** Univariate Cox regression analysis in the development cohort.

Characteristics	HR	95% CI	P
Age>60	1.254	0.661-2.379	0.489
Female	1.253	0.677-2.322	0.473
Deep brain lesions	1.633	0.862-3.094	0.133
Multiple lesions	2.694	1.336-5.431	0.006*
ECOG-PS≥2	2.830	1.506-5.314	0.001*
KPS	0.966	0.951-0.981	<0.001*
LDH>250 IU/L	2.252	1.174-4.320	0.015*
CSF protein>0.45 g/L	1.459	0.781-2.727	0.237
β_2_-MG>2.5 mg/L	3.054	1.463-6.415	0.003*
ALB≥40 g/L	2.793	0.990-7.876	0.052
RDW>0.15	1.963	0.946-4.075	0.070
D-Dimer>0.6 mg/L	1.736	0.904-3.332	0.097
Bcl-2≥60%	2.158	1.159-4.017	0.015*
Bcl-6≥40%	0.819	0.442-1.518	0.527
MUM1≥40%	0.733	0.390-1.377	0.334
C-myc≥40%	2.057	1.041-4.065	0.038*
Ki-67≥80%	1.362	0.665-2.793	0.399
SIRI≥3.3	2.898	1.551-5.415	<0.001*
SII≥97.4	1.579	0.787-3.170	0.199
NLR≥5.1	1.405	0.759-2.601	0.279
PLR≥78.9	0.471	0.216-1.025	0.058
LLR≥220.3	1.924	1.029-3.598	0.041*
βLR≥4.1	3.038	1.402-6.582	0.005*
LMR≥2.7	0.637	0.339-1.197	0.192
PNI≥40	0.394	0.210-0.741	0.004*
AAPR≥0.6	0.653	0.341-1.249	0.198

ECOG-PS, Eastern Cooperative Oncology Group performance status; KPS, Karnofsky performance status; LDH, lactate dehydrogenase; CSF, cerebrospinal fluid; β2-MG, β2-microglobulin; ALB, albumin; RDW, red blood cell volume distribution width; SIRI, systemic inflammation response index; SII, systemic immune inflammation index; NLR, neutrophil-to-lymphocyte ratio; PLR, platelet-to-lymphocyte ratio; LLR, lactate dehydrogenase-to-lymphocyte ratio; βLR, β2-microglobulin-to-lymphocyte ratio; LMR, lymphocyte-to-monocyte ratio; PNI, prognostic nutritional index; AAPR, serum albumin-to-alkaline phosphatase ratio; CI, confidence interval; HR, hazard ratio; *Statistically significant (P<0.05).

**Table 3:** Multivariate Cox regression analysis in the development cohort.

Characteristics	Coefficient	HR	95% CI	P
Lesion number				
Single vs. Multiple	0.937	2.553	1.213-5.377	0.014*
β_2_-MG (mg/L)				
<2.5vs.≥2.5	0.903	2.468	1.158-5.258	0.019*
SIRI				
<3.3vs.≥3.3	0.602	1.826	0.940-3.550	0.076
KPS	-0.028	0.972	0.956-0.988	<0.001*
Statistical analysis of the prognostic model				
Likelihood ratio test				<0.001*
Wald test				<0.001*
Score (log-rank) test				<0.001*

β2-MG, β2-microglobulin; SIRI, systemic inflammation response index; KPS, Karnofsky performance status; CI, confidence interval; HR, hazard ratio; *Statistically significant (P<0.05).

**Figure 3:**
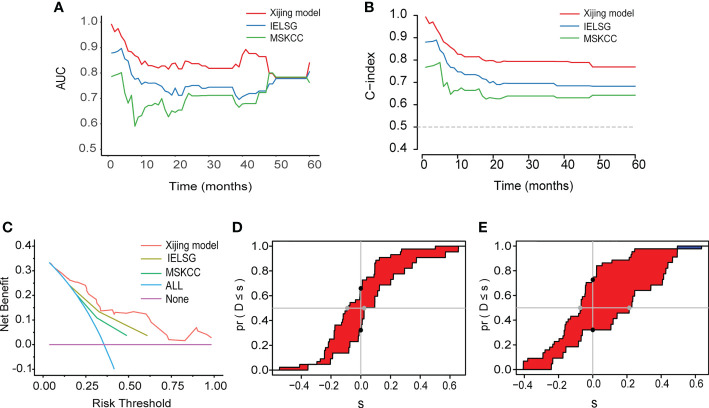
**(A)** The time-dependent AUC of the three models. **(B)** The time-dependent Harrell’s C-index of the three models. **(C)** The DCA was used to estimate clinical usefulness of the three models for predicting 1-year OS. **(D, E)** Compared with the IELSG and MSKCC scores, the IDI and continuous NRI indicated the improvement of prediction ability of the Xijing model. The IDI is the value of the difference in area between red and blue zones. The continuous NRI is the value of the distance between two black dots.

In addition, we compared the Xijing model with the widely used IELSG and MSKCC scores to assess the predictability of the Xijing model. In the development cohort, we took 24 samples as a group, conducted bootstrap with 1000 times resample to draw calibration curves, and evaluated the calibration degree of the Xijing model. The calibration curve of 1-year OS indicated that predictive probability was basically consistent with the actual observed probability (**Figure 2B**). In terms of discrimination, the 1, 2 and 5-year ROC curves of Xijing model are shown in the **Figure 2C**. Both time-dependent AUC and C-index of the Xijing model were overall higher than those of the existing IELSG and MSKCC scores (**Table 4**; **Figures 3A, B**). We also assessed the clinical net benefit of the Xijing model by decision curve analysis (DCA) (**Figure 3C**), which demonstrated that the Xijing model could achieve more positive net benefit and larger area under the decision curve (AUDC) over a wider range of risk threshold than that of IELSG and MSKCC scores (**Table 4**). Moreover, the 2-year and 5-year OS calibration curves and DCA of the Xijing model are presented in the **Supplemental Figure S2**.

**Table 4:** Comprehensive evaluations of different models in the development cohort.

OS	12 months	24 months	60 months
AUC, n (95% CI)			
Xijing Model	0.844 (0.750-0.938)	0.844 (0.740-0.948)	0.841 (0.694-0.989)
IELSG	0.776 (0.676-0.876)	0.747 (0.626-0.867)	0.806 (0.624-0.989)
MSKCC	0.672 (0.539-0.804)	0.720 (0.590-0.850)	0.762 (0.551-0.973)
C-index, n			
Xijing Model	0.814	0.794	0.769
IELSG	0.735	0.696	0.683
MSKCC	0.648	0.636	0.640
Range, n(%)			
Xijing Model	3.63%-98.42%	8.01%-99.99%	13.69%-80.58%
IELSG	15.40%-60.98%	28.32%-54.60%	40.71%-71.07%
MSKCC	19.23%-48.95%	34.25%-73.29%	48.96%-69.68%
AUDC, n			
Xijing Model	0.1288	0.2649	0.3098
IELSG	0.0564	0.1060	0.1423
MSKCC	0.0319	0.0730	0.1248
IDI, n (95% CI), P value			
vs. IELSG	16.5% (1.5%-31.9%) P=0.030^*^	14.4% (1.4%-33.8%) P=0.020^*^	-3.9% (-28.6%-26.2%) P=0.905
vs. MSKCC	24.9% (12.0%-40.9%) P<0.001^*^	19.3% (1.8%-39.7%) P=0.030^*^	1.0% (-24.9%-32%) P=0.826
Continuous NRI, n (95% CI), P value			
vs. IELSG	33.7% (-0.3%-57.5%) P=0.06	23.1% (-5.0%-61.7%) P=0.119	-8.6% (-55.4%-61.2%) P=0.965
vs. MSKCC	40.6% (11.1%-69.0%) P<0.001^*^	38.2% (-5.1%-67.4%) P=0.090	48.8% (-43.2%-76.3%) P=0.577

OS, overall survival; AUC, area under the curve; C-index, Harrell’s concordance index; Range, range of risk threshold to get a positive net benefit in the decision curve analysis; AUDC, area under the decision curve analysis; IDI, integrated discrimination improvement; NRI, net reclassification index; IELSG, International Extranodal Lymphoma Study Group; MSKCC, Memorial Sloan Kettering Cancer Center; *Statistically significant (P<0.05).

We also calculated the IDI and continuous NRI (**Table 4**) of the Xijing model to evaluate whether there was an improvement in the prediction efficiency between the Xijing model and the two existing prognostic scores. Compared with IELSG score, the Xijing model improved the predictive efficiency of 1-year and 2-years OS in PCNSL patients. The model indicated that the IDI of 1-year OS was 16.5% (P=0.03, **Figure 3D**), and the IDI of 2-year OS was 14.4% (P=0.02, **Supplemental Figure S3A**). The difference was significant. The IDI of 5-year OS was -3.9%, no statistical difference (P=0.905, **Supplemental Figure S3C**). However, the 1-year, 2-year, and 5-year continuous NRI of the Xijing model were 33.7%, 23.1%, and -8.6%, respectively, with no statistical difference (P=0.06, 0.119, and 0.965, respectively).

Additionally, compared with MSKCC score, the 1-year and 2-year IDI of the Xijing model was 24.9% (P<0.001, **Figure 3E**) and 19.3% (P=0.03, **Supplemental Figure S3B**), respectively, which indicated the improvement of predictive efficiency for 1-year OS and 2-year OS. However, 5-year IDI was 1%, showing no statistical difference (P=0.826, **Supplemental Figure S3D**). The continuous NRI of 1-year, 2-year and 5-year OS was 40.6%, 38.2% and 48.8%, respectively, and only the continuous NRI of 1-year OS was statistically significant (P<0.001).

### 3.3 Risk stratification of the Xijing model

First of all, we calculated the points of each patient based on nomogram of the Xijing model (**Table S1**), and then used X-tile software to calculate the optimal cut-off value (24) based on all patients’ points. According to the cut-off value, patients were divided into three groups: low-risk group (≤93), medium-risk group (>93 and <141), and high-risk group (≥141).

**Figure 4:**
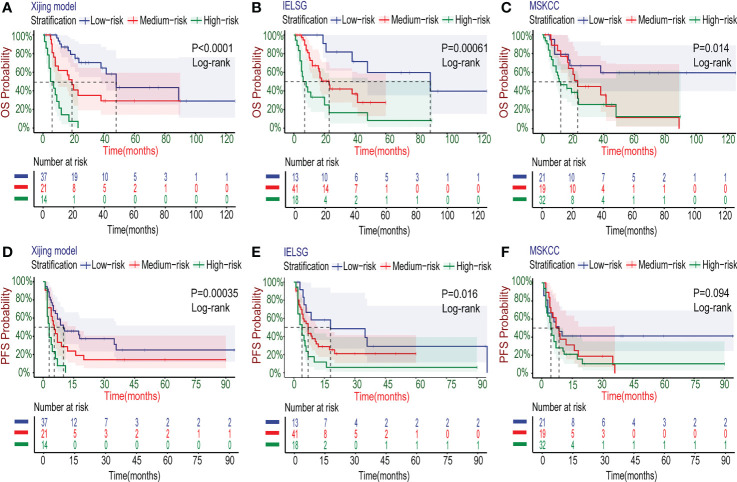
Kaplan–Meier survival curves in the development cohort. **(A-C)** For OS, the PCNSL patients were stratified into three groups by the Xijing Model, IELSG and MSKCC prognostic scores. **(D-F)** The PFS was also classified by above three risk stratifications.

**Figure 5:**
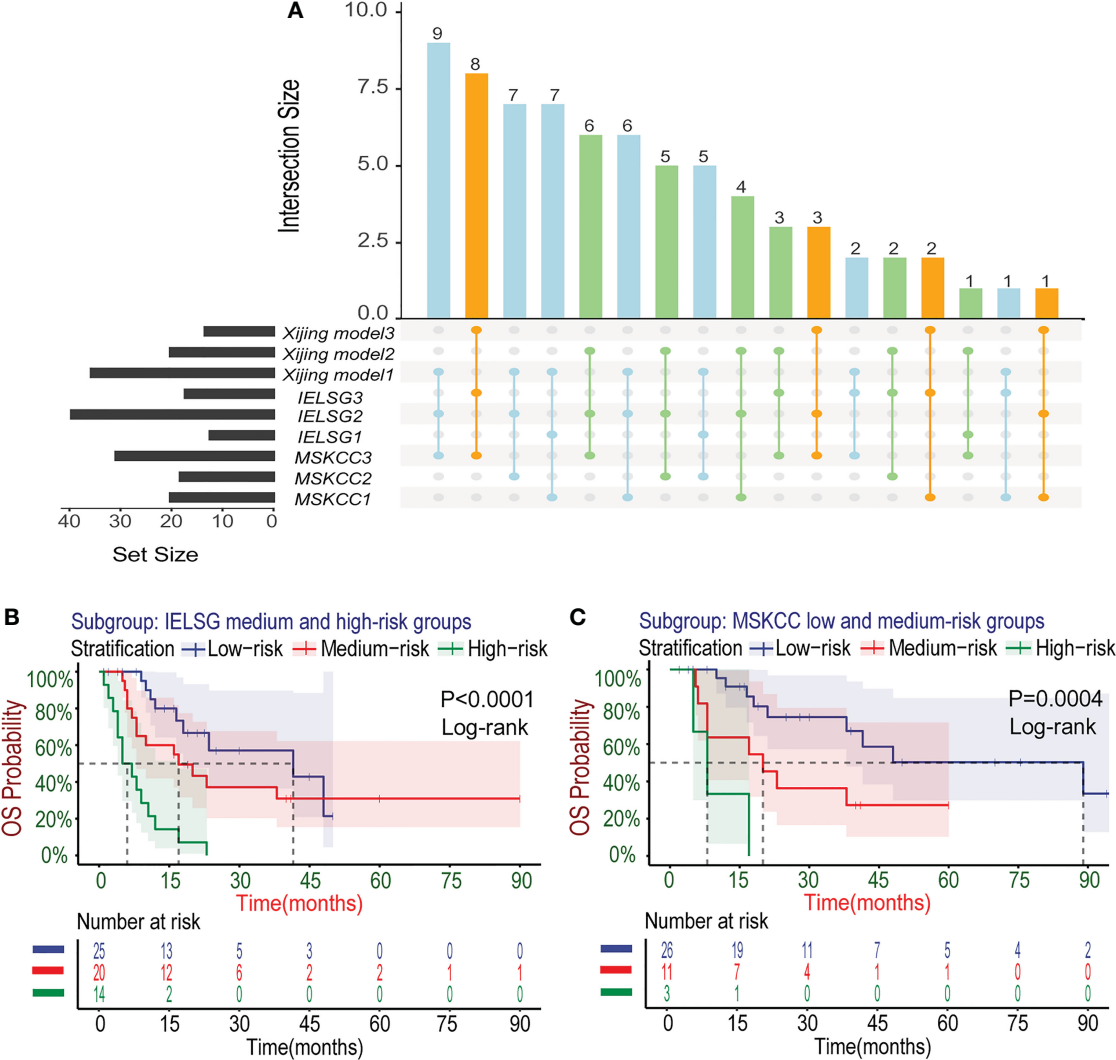
Subgroup analysis about the three risk stratifications in the development cohort. **(A)** The distribution and co-occurrence of the patients respectively classified by the Xijing Model, IELSG and MSKCC scores in the development cohort were shown. Dots and their connected lines indicate that the patients coexisted in different stratifications and the vertical bar graphs reflect the number of these patients. Also, the blue, orange and green dots respectively represent the co-occurrence of the patients who were classified into low, median and high-risk groups by the Xijing Model and other two scores. **(B)** Subgroup analysis with the survival curves for OS in the medium and high-risk groups of IELSG score stratified by the Xiijing model. **(C)** Subgroup analysis with the survival curves for OS in the low and medium-risk groups of MSKCC score stratified by the Xijing model.

In the development cohort (N=72), there were 37(51.4%) patients at low-risk group, 21(29.2%) patients at medium-risk group, and 14(19.4%) patients at high-risk group, with the medium OS of 48, 19 and 5 months and the median PFS of 10, 5.5 and 3 months, respectively (**Table 5**). Both the medium OS and PFS of each group were shorter than that of the corresponding stratification in the IELSG and MSKCC scores, suggesting that the Xijing model may have better performance in finer prognostic stratification. Subsequently, we used the Xijing model, IELSG and MSKCC scores to stratify the patients in the development cohort respectively, and plotted survival curves (**Figures 4A-F**) as well as the distribution and co-occurrence graph of the three stratifications (**Figure 5A**). We found that there existed overlapping survival curves between low and medium-risk groups of the MSKCC score, suggesting poor differentiation of patients in the low and medium-risk groups. Furthermore, the distribution and co-occurrence graph of the patients displayed that there were 54 patients in the medium and high-risk groups of the IELSG score, of which 34 patients were stratified inconsistently with the Xijing model, accounting for 62.7%. While, the stratification of the MSKCC score was inconsistent with that of Xijing model in 19(47.5%) of the 40 patients in the low and medium-risk groups. These above suggest that the Xijing model can further identify specific groups of another two prognostic scores precisely, including the medium and high-risk groups of the IELSG score, as well as the low and medium-risk groups of the MSKCC score. The survival curves shown in **Figures 5B, C** suggest that the specific groups of the IELSG and MSKCC mentioned above can be reclassified into three groups more precisely, and the Log-rank test showed statistical significance between groups (P<0.0001 and P=0.0004).

**Table 5:** The OS and PFS of different stratifications in the development cohort.

Stratification	Median OS (months)	Median PFS (months)
Xijing Model		
Low-risk	48	10
Median-risk	19	5.5
High-risk	5	3
IELSG		
Low-risk	89	18
Median-risk	23	7
High-risk	6	4
MSKCC		
Low-risk	NR	8.5
Median-risk	23	8.5
High-risk	12	4.5

OS, overall survival; PFS, progression-free survival; IELSG, International Extranodal Lymphoma Study Group; MSKCC, Memorial Sloan Kettering Cancer Center; NR, not reached.

### 3.4 Subgroup analysis

**Figure 6:**
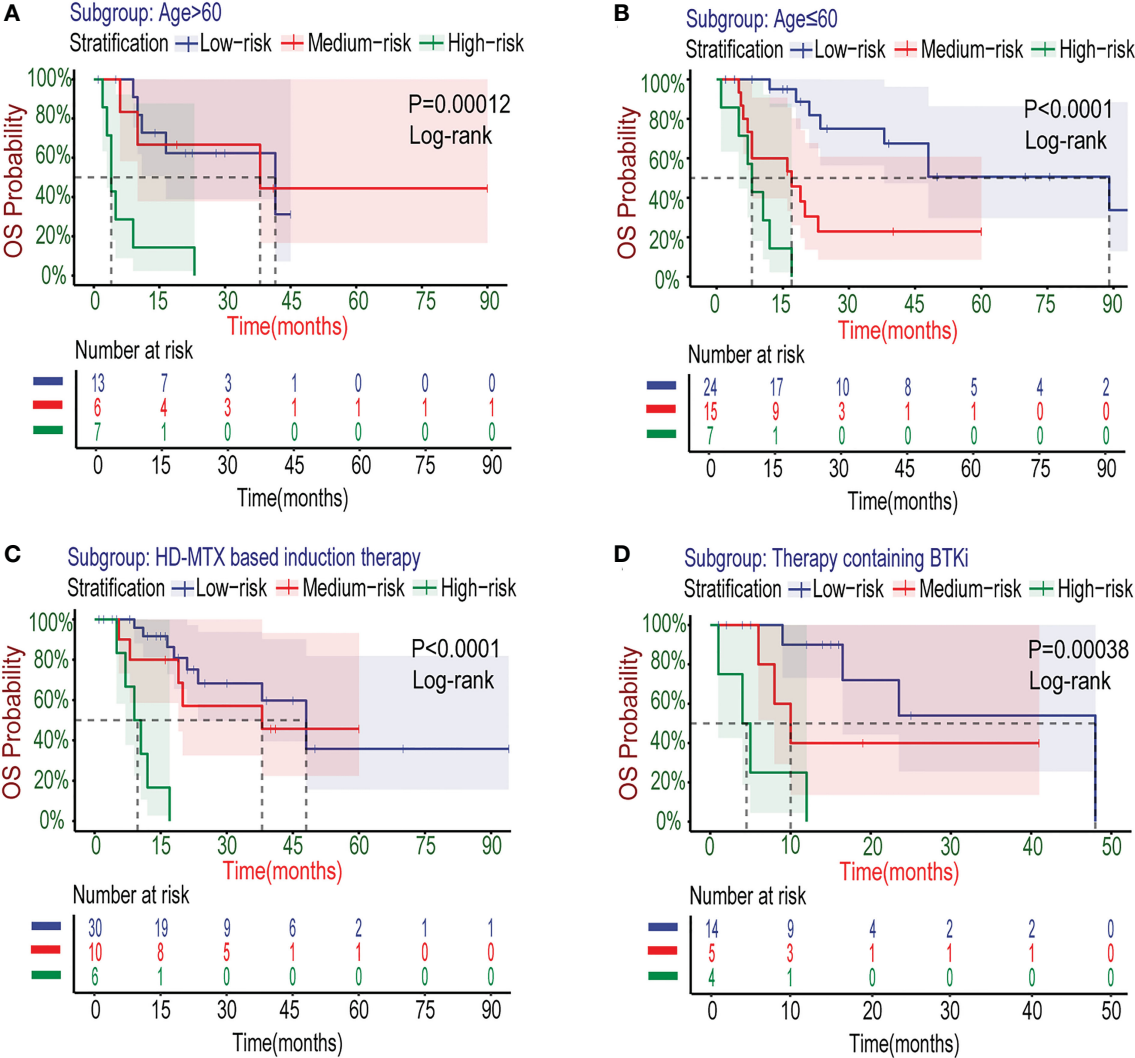
Kaplan–Meier survival curves of specific subgroups stratified by the Xijing Model in the development cohort. The OS curves for subgroups with different characteristics of age **(A, B)** and therapy **(C, D)** were displayed.

Researches have shown that age is one of the important prognostic factors for patients with PCNSL (8–10). In the newly developed Xijing model, we also explored its applicability for specific populations with age>60 years old. The survival curves (**Figures 6A, B**) showed that the Xijing model also had the ability of prognostic prediction in elderly patients with PCNSL.

The treatment for PCNSL is mainly based on standard HD-MTX chemotherapy nowadays. Many patients have already accepted HD-MTX-based induction chemotherapy regimens at the time of admission. Therefore, as a prognostic model, the Xijing model need to be confirmed in the modern combination chemotherapy regimens based on HD-MTX. We collected data from 46 patients with PCNSL after the first course of HD-MTX treatment (**Table S3**), and further explored the stratifying capacity of the Xijing model for these patients by plotting survival curves (**Figure 6C**). We found the model is able to perform risk stratification in the specific subgroup treated with HD-MTX similarly better.

It is worth noting that the Xijing model is equally applicable for PCNSL patients who have been treated with BTKi (detailed treatment regimens including BTKi are shown in **Table S4**) in the development cohort (**Figure 6D**), which suggests that the Xijing model may still perform better in the era of immune targeted therapy for PCNSL, compared to the original scores.

### 3.5 External validation of the Xijing model

**Figure 7:**
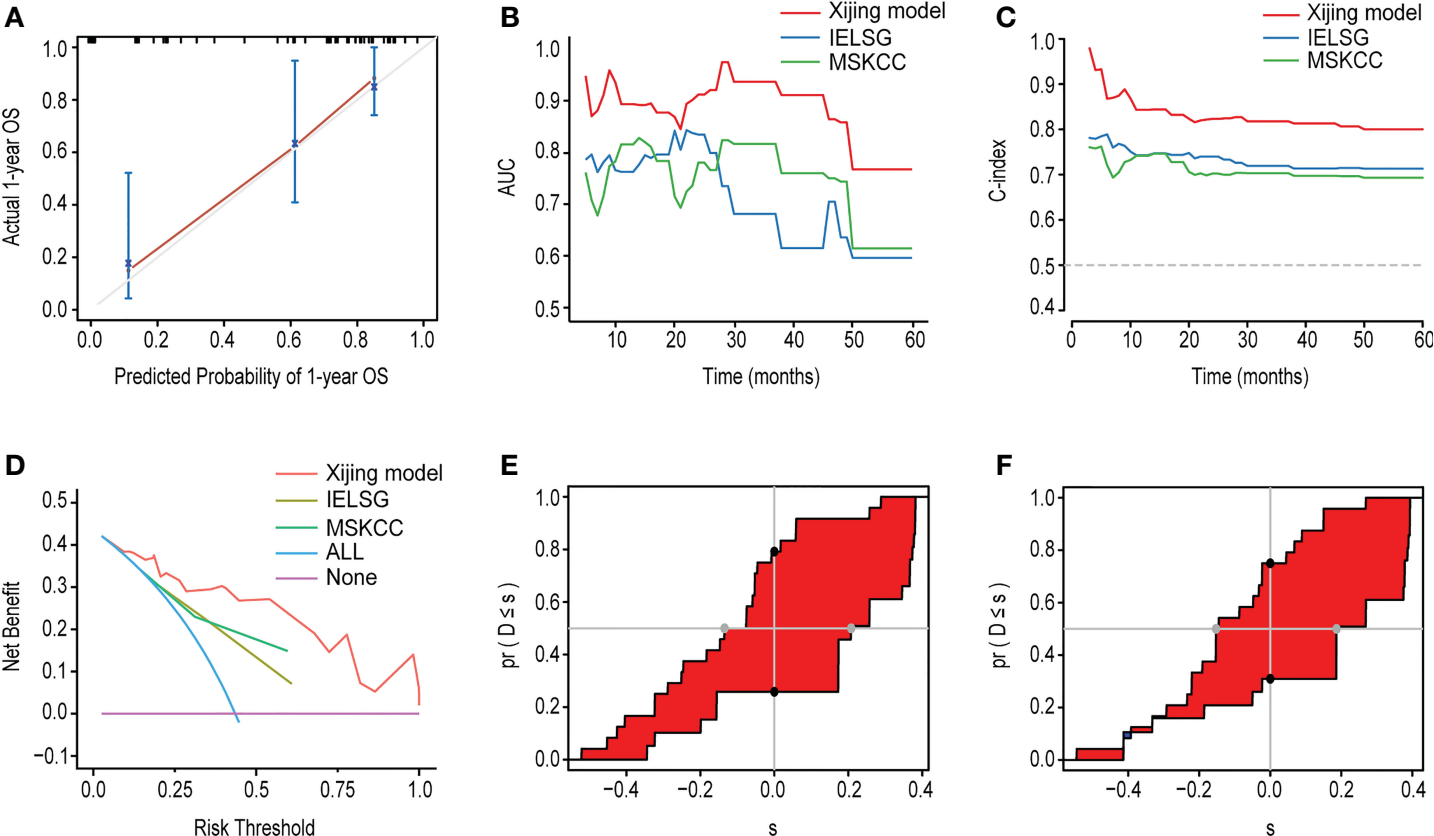
The performance of the Xijing Model, IELSG and MSKCC scores for predicting OS in the validation cohort. **(A)** The time-dependent AUC of the three models. **(B)** The time-dependent Harrell’s C-index of the three models. **(C)** The DCA was used to estimate clinical usefulness of the three models for predicting 1-year OS. **(D)** The improvement in prediction of the Xijing model compared to the IELSG **(E)** and MSKCC **(F)** scores. The IDI is the value of the difference in area between red and blue zones. The continuous NRI is the value of the distance between two black dots.

In the validation cohort, we verified the predictive performance of the Xijing model comprehensively. Specifically, the calibration curves of the Xijing model for 1-year, 2-year and 5-year OS are all close to 45° diagonal line, indicating that the predictive probability of Xijing model is roughly consistent with actual observation results (**Figure 7A**; **Supplemental Figures S4A, B**).

**Figure 8:**
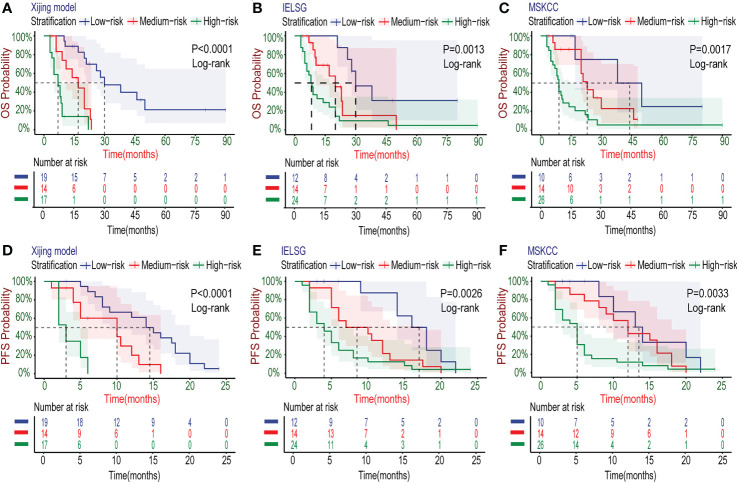
Kaplan–Meier survival curves in the validation cohort. **(A-C)** The OS curves of PCNSL patients stratified by the Xijing Model, IELSG and MSKCC scores. **(D-F)** The PFS curves of PCNSL patients classified by the three risk stratifications.

**Table 6** displays the results of other comprehensive evaluation of the Xijing model in the validation cohort, in which both the time dependence AUC and C-index of Xijing model are overall higher than those of the IELSG and MSKCC scores (**Figures 7B, C**). Besides, the DCA of the Xijing model has a larger net benefit among a wider range of risk thresholds than the other two existing prognostic scores (**Figure 7D**; **Supplemental Figures S4C, D**). According to IDI, the ability of the Xijing model to predict 1-year OS in PCNSL patients was improved, compared to the IELSG and MSKCC prognostic scores (29.6%, P=0.01 and 26.1%, P=0.01, **Figures 7E, F**). Particularly, the Xijing model still has a tendency to perform better than the MSKCC score in predicting 2-year OS (18%, P=0.06, **Supplemental Figure S5B**). But compared with the IELSG score, there was no statistical difference about the predictive improvement shown by 2-year IDI of the Xijing model (10%, P=0.398, **Supplemental Figure S5A**). As for the continuous NRI, the Xijing model showed an improvement in 1-year and 2-year OS prediction compared with MSKCC score (44.1%, P=0.04 and 48.3%, P=0.05), and also showed a better trend in 1-year OS prediction compared with IELSG score (53.3%, P=0.07). However, there is no statistical difference in the improvement of continuous NRI in 2-year OS prediction (31.4%, P=0.199). For the 5-year OS prediction of PCNSL patients, there was no statistical difference in the improvement of prediction efficiency of IDI and continuous NRI in the Xijing model (**Supplemental Figures S5C, D**). Finally, we used the nomogram mentioned above to calculate the total points of each patient in the validation cohort and divided those patients into three groups according to the risk-stratification criteria of the Xijing model. The groups were as follows: 19(38%) patients in the low-risk group, 14(28%) patients in the medium-risk group, and 17(34%) patients in the high-risk group, with median OS of 30 months, 17 months, and 7 months and median PFS of 14 months, 10 months and 3 months, respectively. Subsequently, the OS and PFS survival curves of the validation cohort were plotted (**Figures 8A-F**) with statistical difference existing in both the Xijing model and the above mentioned two prognostic scores by the Log-rank test.

**Table 6:** Comprehensive evaluations of different models in the validation cohort.

OS	12 months	24 months	60 months
AUC, n (95% CI)			
Xijing Model	0.895 (0.801-0.989)	0.913 (0.829-0.997)	0.769 (0.623-0.914)
IELSG	0.764 (0.641-0.887)	0.836 (0.675-0.997)	0.598 (0.198-0.998)
MSKCC	0.817 (0.709-0.924)	0.782 (0.624-0.940)	0.616 (0.236-0.996)
C-index, n			
Xijing Model	0.843	0.823	0.80
IELSG	0.743	0.740	0.713
MSKCC	0.742	0.702	0.693
Range, n(%)			
Xijing Model	2.54%-100%	8.21%-100%	22.82%-100%
IELSG	34.17%-64.90%	37.36%-90.56%	58.97%-86.48%
MSKCC	14.25%-59.60%	29.89%-87.68%	53.82%-84.68%
AUDC, n			
Xijing Model	0.2367	0.4710	0.5486
IELSG	0.0865	0.1988	0.1864
MSKCC	0.1022	0.1974	0.1871
IDI, n (95% CI), P value			
vs. IELSG	29.6% (5.20%-49.8%) P=0.01^*^	10.0% (-12.9%-32.6%) P=0.398	-5.5% (-34.0%-40.1%) P=0.915
vs. MSKCC	26.1% (3.7%-44.9%) P=0.01^*^	18.0% (-1.1%-38.9%) p=0.06	-8.8% (-44.3%-39.8%) P=0.796
Continuous NRI, n (95% CI), P value			
vs. IELSG	53.3% (-1.8%-79.0%) P=0.07	31.4% (-1.0%-57.2%) P=0.199	25.4% (-42.0%-78.9%) P=0.557
vs. MSKCC	44.1% (1.0%-79.4%) P=0.04^*^	48.3% (0%-71.9%) P=0.05	20.5% (-46.3%-82.4%) P=0.627

OS, overall survival; AUC, area under the curve; C-index, Harrell’s concordance index; Range, range of risk threshold to get a positive net benefit in the decision curve analysis; AUDC, area under the decision curve analysis; IDI, integrated discrimination improvement; NRI, net reclassification index; IELSG, International Extranodal Lymphoma Study Group; MSKCC, Memorial Sloan Kettering Cancer Center; *Statistically significant (P<0.05).

## 4 Discussion

PCNSL refers to a class of rare malignancies originating in the CNS without peripheral involvement. The annual incidence of PCNSL has increased in recent decades, which is about 0.48/100,000 (25). More than 80% of patients with PCNSL have intracranial lesions, and only a few have leptomeninges and eyes involvement (26). The clinical symptoms of PCNSL mostly include consciousness disturbance, headache, hemiplegia, epilepsy, aphasia, and visual abnormalities, which are easy to be misdiagnosed because of specificity lacking (2). At present, there is no clear and unified standard for the treatment of PCNSL, and the major treatments include MTX-based combined chemotherapy, surgical resection, whole brain radiation therapy, etc. (27). In recent years, with the introduction of new strategies, such as molecular-targeted drugs, autologous hematopoietic stem cell transplantation (AHSCT) and chimeric antigen receptor T-cell immunotherapy (CAR-T), though the survival rate of PCNSL patients has improved, the overall prognosis of PCNSL is still unsatisfactory. It has been reported that the 5-year OS rate of PCNSL patients is only 15-30%, which undoubtedly increases the economic burden on both patients and society (10, 14, 28).Therefore, a reliable prognostic prediction model which can stratify accurately and guide clinical decisions is of particular importance for patients with PCNSL.

The existing IELSG and MSKCC prognostic scores are widely used in clinical practice, but they both have their own limitations. Although the research that developed the IELSG score was based on multi-center and had a large sample size (N=378), data on LDH and CSF proteins were missing in 2/3 of the samples (8). Besides, we found that CSF protein is usually difficult to obtain in clinical practice due to the contraindications of lumbar puncture, the non-cooperation of patients and unnecessary of examination. Therefore, it is difficult for some patients to effectively predict the prognosis by IELSG score, which also limits the clinical application of IELSG score (29–31). As for the MSKCC score, though external validation of its predictive validity has been confirmed in the original study, there still be bias in the risk stratification based on the two variables of age and KPS only. Some relevant studies have not found a strong association between the MSKCC score and the OS of patients with PCNSL (32, 33), which caused controversy over the reliability of the MSKCC score. Thus, some researches attempted to improve the predictive capacity of the MSKCC score by adding some prognosis-related factors, such as SII and TBIL (34, 35). Unfortunately, all the subjects of the improved MSKCC score were from single-center and received high-dose MTX chemotherapy, and some of them received glucocorticoids before diagnosis, which may affect prognostic prediction and lead to bias. In addition, the lack of external validation also limits the universality of the improved MSKCC score.

Based on 122 patients with PCNSL from two medical centers in China, our research has developed a novel and simple prognostic model and plot the nomogram. Considering that the median OS of the development cohort was 21 months (roughly 2 years) and most patients achieved primary end event within 2 years (2-year survival probability 45.6%), we paid more attention to 2-year OS rather than the usual 3-year OS, which was also similar to a Singaporean retrospective study by Lo YT et al. (36). Thus, the Xijing model is used to predict the 1,2 and 5-year OS for PCNSL. First of all, we screened out six prognosis-related candidate variables by univariate Cox regression analysis and Lasso analysis, including age, Bcl-2, the number of lesions, KPS, β2-MG and SIRI. However, Bcl-2 is only expressed on the cell surface of B-cell lymphomas, and the pathological types of PCNSL include peripheral T-cell lymphomas in addition to B-cell lymphomas. Besides, the evaluation of Bcl-2 IHC positive results depends on the judgment of professional pathologists, which is greatly affected by clinical experience and other personal factors and is prone to bias. Therefore, Bcl-2 is not a suitable candidate variable for all patients with PCNSL. As for age, with the development of treatment, the OS of PCNSL patients has been improved, and the influence of age on the prognosis of PCNSL has a downward trend. To sum up, given the clinical relevance and statistical significance, the remaining four variables, the number of lesions, β2-MG, KPS and SIRI, were incorporated eventually to develop the prognostic model and ensure parsimony of the final model. Thus, the Xijing model predicts 1,2 and 5-year survival probability for PCNSL.

Then, we drew calibration curves, DCA and calculated time-dependent AUC, time-dependent C-index, IDI and continuous NRI, and compared the Xijing model with two widely used existing scores to evaluate the prediction capability of the Xijing model. The results of our research show that the above evaluation indicators of the Xijing model have an overall improvement, compared with the IELSG and MSKCC scores. The Xijing model performs better in terms of discrimination, calibration, clinical net benefit and predictive efficiency, and has greater prognostic prediction value for PCNSL patients. We also used the nomogram of Xijing model to score the patients in the development cohort, and divided them into low-risk (≤93), medium-risk (>93 and <141) and high-risk group (≥141) by the cut-off value. The survival curves were drawn and statistically tested, and there were statistical differences among the groups. Finally, we completed the external validation of the Xijing model in the validation cohort.

In addition to the improvement of predictive capacity, compared with the two existing prognostic scores, the advantages of the Xijing model are that the four variables it contains are more easily to be obtained in clinical practice and the process of prognostic assessment is visual. Among them, the number of lesions can be achieved by imaging examination and reflects the extent of tumor involvement. KPS is generally completed at the time of admission assessment, which can easily reflect the patients’ physical condition. β2-MG, which reflects the patient’s tumor burden, can be obtained by peripheral blood tests (37–39), while SIRI can be calculated by complete blood count. Our study has shown that the four variables of Xijing model are all related to the prognosis of PCNSL patients. Compared with the only two variables of MSKCC score, a more comprehensive judgment can be made from the four aspects of the number of lesions, tumor burden, systemic inflammatory response and physical condition. It is worth mentioning that SIRI is an emerging indicator of systemic inflammation in recent years. More and more evidence suggesting that tumor-related inflammatory response promotes the proliferation, invasion and metastasis of tumor cells (40–42), and more studies have confirmed that SIRI is an independent prognostic risk factor for kinds of malignant tumors, including breast cancer, hepatocellular carcinoma, glioblastoma, pancreatic cancer as well as PCNSL (7, 43–47).

Xijing model can more comprehensively predict the 1-year, 2-year and 5-year OS probability of PCNSL patients from four aspects: imaging findings, tumor burden, systemic inflammatory response index and comprehensive physical condition. In addition, the Xijing model can further stratify the medium and high-risk groups of IELSG as well as the low and medium-risk groups of MSKCC, indicating that the Xijing model performs better on detailed stratification and accurate prediction for patients with PCNSL. Similarly, the elderly with PCNSL and specific populations who have accepted HD-MTX or BTKi treatment can also be stratified by the Xijing model, which validates the utility of the Xijing model in specific subgroups. Finally, due to the fact that the source of samples for the development and validation cohort were inevitably different, the Xijing model showed good predictive ability in both cohorts, suggesting the universality of the Xijing model.

However, our model still exists some limitations. First, PCNSL is rare, and it is difficult to recruit a large number of patients in clinical practice. Therefore, the sample size for the new model is small, and a larger sample size from multiple medical centers is needed for validation in the future. Second, the study was based on a Chinese population, which may affect how the Xijing model performs in other populations.

## 5 Conclusion

In summary, we developed a new PCNSL prognostic model based on real-world data and visualized it by nomogram. The variables in the model are easy to obtain and strongly practical. The validation results demonstrate that the Xijing model has better prediction ability, universality, and higher clinical application value.

## Data availability statement

The raw data supporting the conclusions of this article will be made available by the authors, without undue reservation.

## Ethics statement

The studies involving human participants were reviewed and approved by the Ethics Committee of the Xijing and Tangdu hospital. The patients/participants provided their written informed consent to participate in this study.

## Author contributions

ZWu and CW conceived the present study, participated in its methodology design, drafted the manuscript and interpreted the data. RL, GG, MH and ZWa conceptualized the study and participated in methodology design. JW, XD, NZ, JG and YZ enrolled the patients. YaL, ZL, ML, SW, BW, NY and YeL acquired the data. Data analysis was performed by ZWu and CW. All authors contributed to the article and approved the submitted version.
