# Supplementary material for: A novel inflammation-related prognostic model for predicting the overall survival of primary central nervous system lymphoma: A real-world data analysis
Source: Front Oncol. 2023 Mar 28;13:1104425. doi: 10.3389/fonc.2023.1104425 (PMC10086228; doi:10.3389/fonc.2023.1104425)
Supplement: Supplementary file 1 [file DataSheet_1.pdf]

Supplementary Material

The R packages used in the process

The R packages involved are shown as following: (1) ‘lattice’, ‘MASS’, ‘nnet’, ‘foreign’ and ‘mice’ are applied for Multiple imputation. (2) ‘survival’ and ‘glmnet’ are applied for Lasso analysis. (3) ‘survival’, ‘rms’ and ‘nomogramFormula’ are applied for univariate and multivariate Cox regression analysis, as well as nomogram and point calculation based on the prognostic model. (4) ‘rms’ is used to plot calibration curves. (5) ‘survival’ and ‘timeROC’ are used to plot ROC. (6) ‘survival’, ‘riskRegression’, ‘ggplot2’, ‘ggprism’, ‘rms’ and ‘pec’ are used to compare the time-dependent AUC and C-index between the Xijing model and the two original prognostic scores. (7) ‘rms’, ‘ggDCA’, ‘survival’, ‘ggplot’ and ‘ggprism’ are used for DCA. (8) ‘survival’ and ‘survIDINRI’ are utilized to calculate IDI and continuous NRI. (9) ‘survminer’ and ‘survival’ are utilized for survival analysis.

Table S1. Points for each constructed variables in the nomogram

Variable	Point	Variable	Point
**Lesion Number**		**KPS**	
Single	0	10	100
Multiple	41	20	88
**β2-MG (g/L)**		30	75
≤2.5	0	40	63
>2.5	40	50	50
**SIRI**		60	38
<3.3	0	70	25
≥3.3	26	80	12
—	—	90	0

Table S2. The 1, 2 and 5-year survival probability based on total points of the nomogram

Survival Probability	Total Points		
	1-year	2-year	5-year
0.1	181	146	121
0.2	166	130	105
0.3	153	117	92
0.4	141	105	80
0.5	129	93	68
0.6	115	79	55
0.7	100	64	39
0.8	79	43	18
0.9	46	10	—

Table S3. Characteristics of subgroup received HD-MTX

Constructed variables	Subgroup received HD-MTX
**Multiple lesions, n(%)**	25(54.3)
**Median KPS(IQR)**	60(60-70)
**β_2-MG>2.5 mg/L, n(%)**	20(43.5)
**SIRI≥3.3, n(%)**	15(32.46)

Constructed variables refer to the variables contained in the Xijing model.

Table S4. Regimens including BTKi

BTKi	Usage	n
**Ibrutinib**	560mg qd	5
**Zebutinib**	160mg bid	8
**Obutinib**	150mg qd	10

BTKi, Bruton's tyrosine kinase inhibitors.

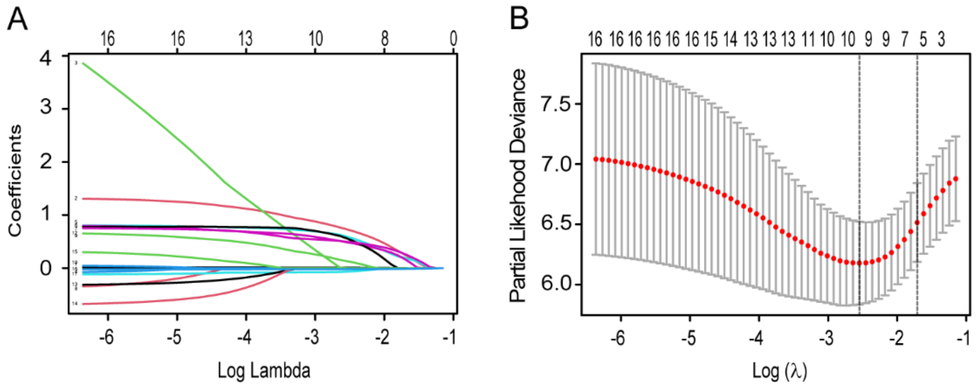

Figure S1. The results of Lasso analysis. (A) Coefficients in Lasso analysis. (B) 1000 bootstrap resamples by Lasso analysis

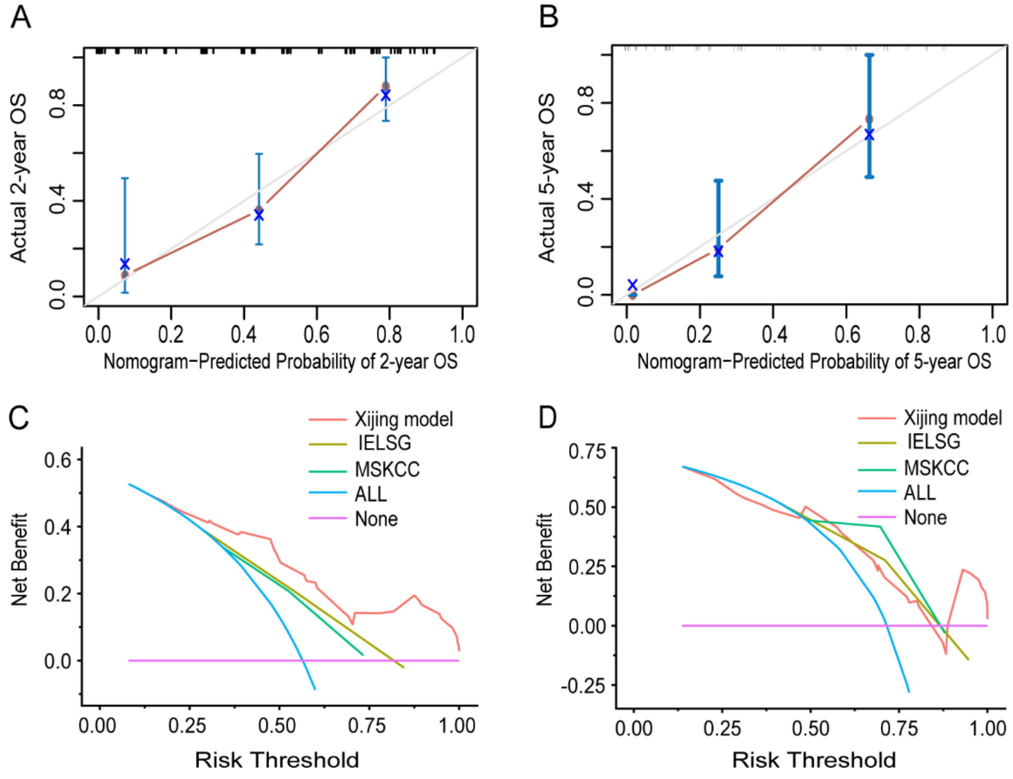

Figure S2. Calibration curve and DCA of the Xijing model in the development cohort. (A, B) The calibration curve of the Xijing model for 2-year and 5-year OS. (C, D) The 2-year and 5-year DCA of the Xijing model.

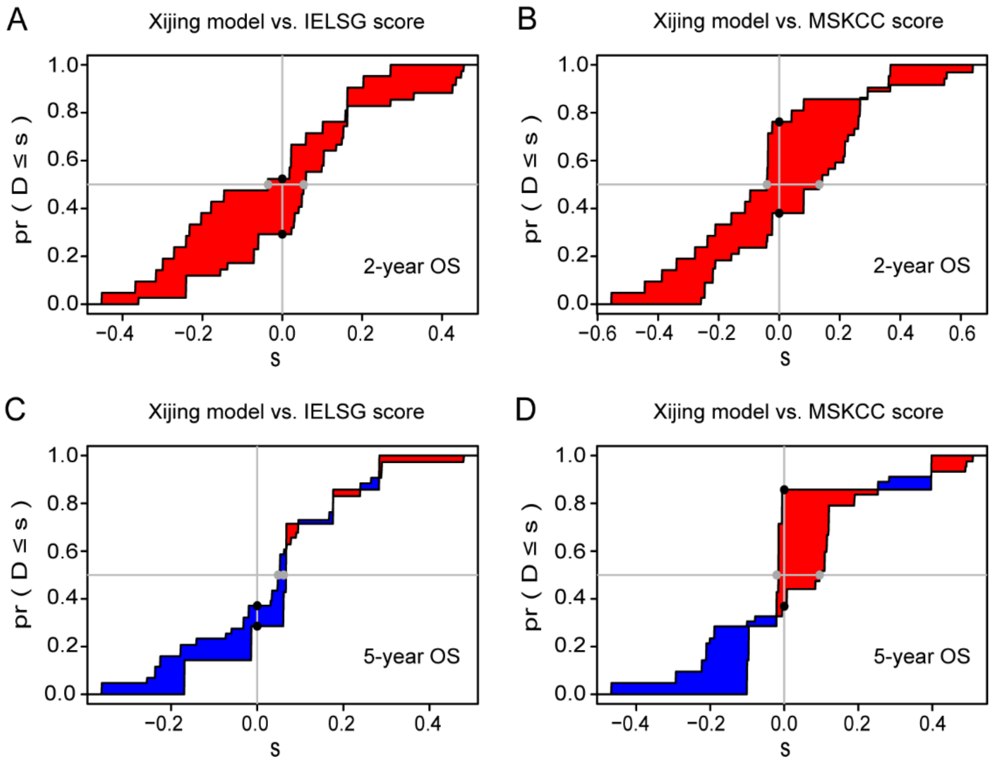

Figure S3. Compared the Xijing model with the IELSG score (A, C) and the MSKCC score (B, D), the improvement of predictive capacity for 2-year and 5-year OS according to IDI and continuous NRI in the development cohort.

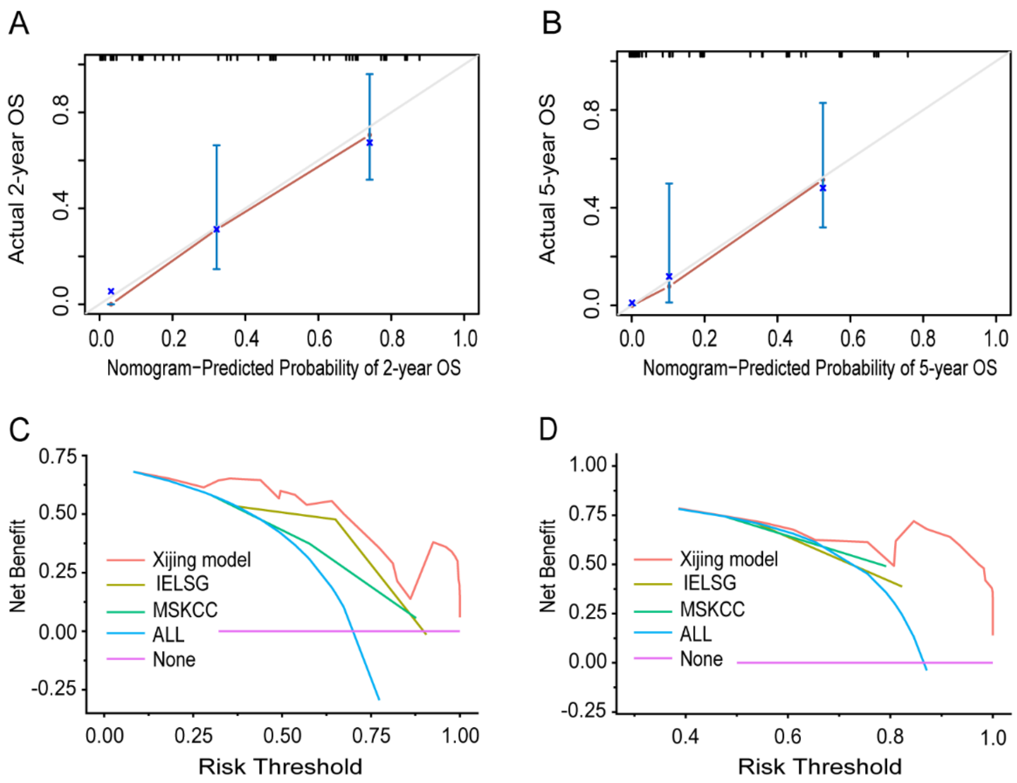

Figure S4. Calibration curve and DCA of the Xijing model in the validation cohort. (A, B) The calibration curve of the Xijing model for 2-year and 5-year OS. (C, D) The 2-year and 5-year DCA of the Xijing model.

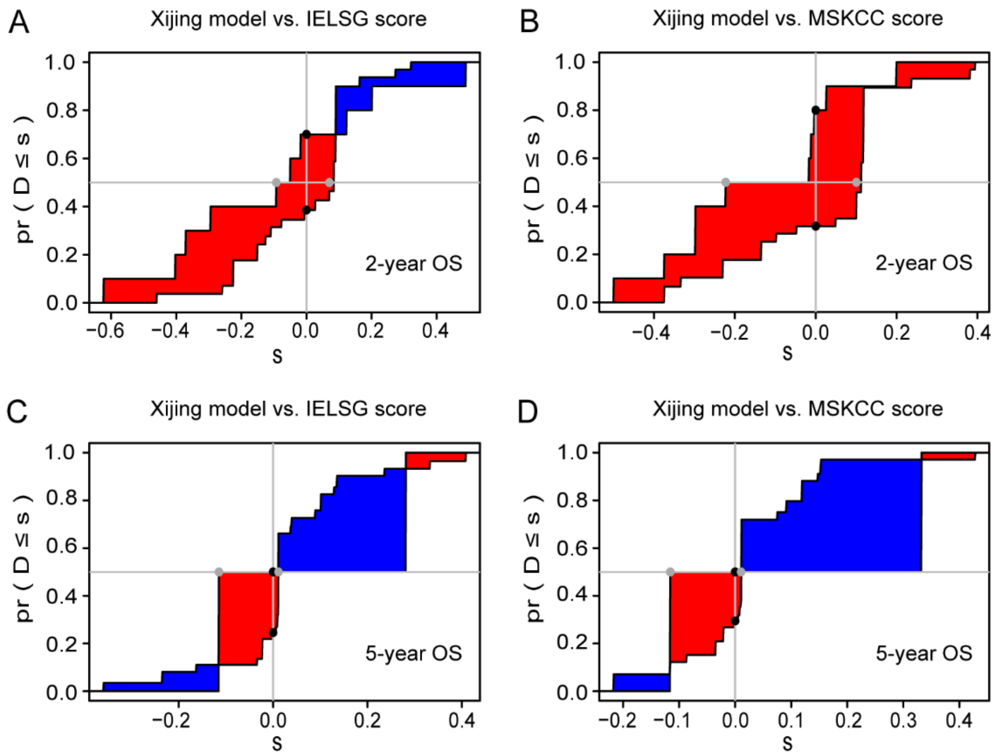

Figure S5. Compared the Xijing model with the IELSG score (A, C) and the MSKCC score (B, D), the improvement of predictive capacity for 2-year and 5-year OS according to IDI and continuous NRI in the validation cohort.
